# Time for a change, time to say thank you

**DOI:** 10.1186/s12983-025-00558-2

**Published:** 2025-02-28

**Authors:** Ulrich Technau

**Affiliations:** Department for Neurosciences and Developmental Biology, Division of Molecular Evolution and Development, Research platform Single Cell Regulation of Stem Cells, Faculty of Life Sciences, University of Vienna, University of Vienna Biology Building (UBB), Djerassiplatz 1, 1030 Wien, Austria

It is hard to imagine our journal *Frontiers in Zoology* without Jürgen Heinze, who has been the Co-Editor in Chief since 2004, when he and Diethard Tautz founded the journal as the official journal of the German Zoological Society (DZG). The goal and mission was and is to establish a leading journal in the field of Zoology and indeed since the beginning, *Frontiers in Zoology* has been mostly ranked among the top 10 journals in the category Zoology. To keep this high quality is to a large extent the merit of Jürgen Heinze. However, with his retirement, Jürgen decided to step down from his role as a Co-Editor in Chief for *Frontiers in Zoology*. Effective with January 1, 2025, he hands over the torch to Angelika Stollewerk, for many years herself a member of the Editorial Board of *Frontiers in Zoology.* Since 2014, when I took over the co-Editior in Chief position from Diethard Tautz, I have been working with Jürgen and got to know him a vigilant, careful and thoughtful Editor, who always kept an eye on the whole journal, somebody who was constantly looking after and promoting the journal. Therefore, also in the name of the board of the DZG, I would like to take this opportunity to express our enormous gratitude to Jürgen Heinze for serving as an Editor in Chief for more than 20 years.

As an Editor, one has to take many decisions about manuscripts and there is hardly any field that is more diverse than Zoology. His work as an Editor was always guided by fostering this breadth of topics in Zoology, yet foremost by applying high standards in scientific quality and ethics. He followed the same high standards in his own scientific work. Jürgen was trained as a behavioral and evolutionary entomologist at the TU Darmstadt, a topic he remained faithful to throughout his whole career. He did a postdoc with E.O Wilson, followed by a research fellow (“University assistant”) in the lab of Bert Hölldobler, two “giants” in the field of ant biology. In 1996 he became Professor at the University of Nürnberg-Erlangen and moved to a full professorship at the University of Regensburg in 2000. He has been a leader in the field of social insects, specializing on cooperation and conflict in ant populations. Besides his continuous work as Editor-in-Chief of *Frontiers in Zoology* he was active in several boards of the German Science Foundation and the “Wissenschaftsrat”, the Science Council of the German government. He is elected member of several academies, including the National German Academy of Science, Leopoldina and was awarded the Karl von Frisch Medal of the DZG. All these activities and honors reflect the deep appreciation and recognition he has received from the scientific community. We cordially thank Jürgen for his tremendous work and the service to the Science community, for being an advocate for Zoology and for developing *Frontiers in Zoology* as one of the top journals in the field of Zoology.

Angelika Stollewerk has taken over as co-Editor-in-Chief in January 2025. She is an expert in the evolution of arthropod nervous systems. She studied the development and interaction of neural cell types first in traditional model organisms before moving into the field of Evolutionary Developmental Biology. She investigates the roles of neural genes in representatives of all arthropod groups to understand how the diversity of nervous systems and sensory organs have evolved and has made major contribution to our knowledge of the neural development and of the evolutionary pathway of neurogenesis in arthropods. For her early work on spiders and myriapods, she received the Walther Arendt award of the DZG in 2005. After her habilitation with Diethard Tautz at the University of Cologne, Angelika was awarded a Heisenberg-Stipendium which she used for research stays at the Department of Zoology in Cambridge, UK, and at the Institute of Genetics at the University of Mainz to expand the range of her research. She then took on the position of Associate Professor at Queen Mary University of London.

Having extensive experience in editorial tasks, Angelika is well prepared to follow in Jürgen’s (big) footsteps and we are looking forward to a new chapter in the journey of *Frontiers in Zoology*.

